# A novel silicone derivative of natural osalmid (DCZ0858) induces apoptosis and cell cycle arrest in diffuse large B-cell lymphoma via the JAK2/STAT3 pathway

**DOI:** 10.1038/s41392-020-0123-0

**Published:** 2020-04-01

**Authors:** Kang Lu, Bo Li, Hui Zhang, Zhijian Xu, Dongliang Song, Lu Gao, Haiguo Sun, Liping Li, Yingcong Wang, Qilin Feng, Gege Chen, Liangning Hu, Rong Wei, Yongsheng Xie, Dandan Yu, Xiaosong Wu, Weiliang Zhu, Jumei Shi

**Affiliations:** 1 Department of Hematology, Shanghai Tenth People’s Hospital, Tongji University, School of Medicine, 200072 Shanghai, China; 2 Medical School of Nantong University, 226001 Nantong, China; 3 CAS Key Laboratory of Receptor Research; Drug Discovery and Design Center, Shanghai Institute of Materia Medica, Chinese Academy of Sciences, 201203 Shanghai, China

**Keywords:** Drug development, Target identification

## Abstract

Diffuse large B-cell lymphoma (DLBCL) is a highly heterogeneous malignant tumor characterized by diffuse growth. DCZ0858 is a novel small molecule with excellent antitumor effects in DLBCL. This study explored in depth the inhibitory effect of DCZ0858 on DLBCL cell lines. Cell Counting Kit-8 (CCK-8) and plate colony formation assays were used to evaluate cell proliferation levels. Flow cytometry was employed to analyze apoptosis and the cell cycle, and western blotting was used to quantify the expression of cell cycle regulators. The results indicated that DCZ0858 inhibited cell growth in a concentration-dependent and time-dependent manner while inducing no significant toxicity in normal cells. Moreover, DCZ0858 initiated cell apoptosis via both internal and external apoptotic pathways. DCZ0858 also induced cell cycle arrest in the G0/G1 phase, thereby controlling cell proliferation. Further investigation of the molecular mechanism showed that the JAK2/STAT3 pathway was involved in the DCZ0858-mediated antitumor effects and that JAK2 was the key target for DCZ0858 treatment. Knockdown of JAK2 partly weakened the DCZ0858-mediated antitumor effect in DLBCL cells, while JAK2 overexpression strengthened the effect of DCZ0858 in DLBCL cells. Moreover, a similar antitumor effect was observed for DCZ0858 and the JAK2 inhibitor ruxolitinib, and combining the two could significantly enhance cancer-suppressive signaling. Tumor xenograft models showed that DCZ0858 inhibited tumor growth in vivo and had low toxicity in important organs, findings that were consistent with the in vitro data. In summary, DCZ0858 is a promising drug for the treatment of DLBCL.

## Introduction

Non-Hodgkin lymphoma (NHL), the most common malignancy of the blood system, is one of the 10 leading cancers in terms of incidence and mortality in the United States, with no significant differences in these values between men and women.^1^ Diffuse large B-cell lymphoma (DLBCL) is the most common NHL subtype and includes two major molecular classes, as assessed by gene expression profiling: germinal center B cell-like (GCB) and activated B cell-like (ABC) DLBCL.^2^ For nearly two decades, the standard combination immunochemotherapy treatment, R-CHOP (including rituximab, cyclophosphamide, doxorubicin, vincristine, and prednisone), has greatly improved the prognosis of DLBCL patients, showing a complete response rate of ~80%.^3^ However, because of the heterogeneity of DLBCL, a portion of patients (with double-hit or double-protein-expression lymphoma) do not respond to R-CHOP and have an unsatisfactory outcome, highlighting the limits of standard cytotoxic therapy.^4^ Thus, for this subset of patients, alternative strategies should be explored. For this reason, it would be of great benefit to explore the molecular heterogeneity of DLBCL and investigate novel targeted agents based on the pathological mechanism.

The Janus kinase 2 (JAK2)/signal transducer and activator of transcription 3 (STAT3)-signaling pathway has been widely reported to directly or indirectly participate in the malignant progression of multiple tumors. STAT3 is a DNA-binding transcription factor that can translocate into the cell nucleus and bind to interferon-gamma-activated sequences (GAS) in target gene promoters, thus regulating gene transcription.^5^ In tumor cells, STAT3 is frequently activated, partly due to the aberrant activity of its upstream factors, such as JAK, and constitutive STAT3 activation has been frequently linked to malignant cancer and unfavorable prognoses.^6^ For example, polymorphisms in STAT3 are significantly associated with lymphoma risk, and STAT3 activation is strongly associated with poor clinical outcomes for DLBCL patients who received R-CHOP treatment.^7,8^ Notably, inhibiting STAT3 directly via STAT3 knockdown or indirectly using JAK inhibitors could result in decreased cell proliferation and increased apoptosis in ABC tumor cell lines.^9,10^

In the current study, we investigated the biological effects of DCZ0858, a newly synthesized organosilicon compound, on DLBCL both in vivo and in vitro. Functional experiments showed that DCZ0858 had a tumor-suppressive effect on DLBCL cells, mainly through cell proliferation inhibition, apoptosis induction, and cell cycle arrest via the JAK2/STAT3-signaling pathway. In addition, DCZ0858 effectively inhibited tumorigenesis in a mouse xenograft model. Our findings suggest that DCZ0858 has great potential as a novel therapeutic agent for DLBCL.

## Results

### DCZ0858 inhibits DLBCL cell growth and proliferation

**Fig. 1:**
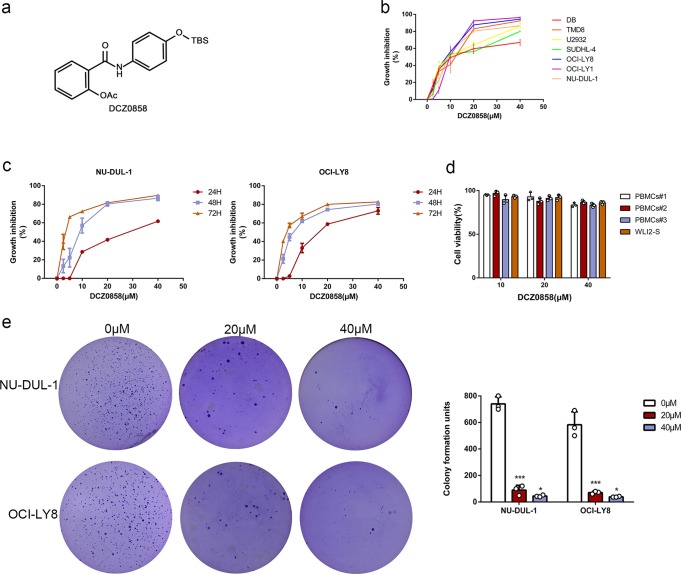
DCZ0858 inhibits DLBCL cell growth and proliferation. **a** Chemical structure of DCZ0858. **b** DLBCL cell lines (DB, TMD8, U2932, SUDHL-4, OCI-LY8, OCI-LY1, and NU-DUL-1) were treated with DCZ0858 at the indicated concentrations for 48 h. **c** NU-DUL-1 and OCI-LY8 were treated with DCZ0858 (2.5–40 μM) and processed at the indicated times. **d** PBMCs from three healthy volunteers and human B lymphocytes (WIL2-S) were treated with DCZ0858(10, 20, 40 μM) for 48 h. Cell viability was analyzed by CCK-8 assay. **e** Cell clone colonies formed by the NU-DUL-1 and OCI-LY8 cells treated with DCZ0858 (0, 20, and 40 μM); the colonies in each well were quantified (*n* = 3; *P < 0.05 and ***P < 0.001)

Clinically, osalmid is a medicine used for treating acute and chronic cholecystitis and gallstone disease that simultaneously has the effect of ameliorating jaundice. Previously published literature reported that osalmid is a potential ribonucleotide reductase small subunit M2-targeting compound and possesses potent activity against a 3TC-resistant hepatitis B virus strain.^11^ In our previous study, we also found that a compound that consisted of oxophenamide and pterostilbene showed excellent antitumor effects on multiple myeloma.^12^ As shown in Fig. 1a, DCZ0858 is a novel silicone derivative of natural osalmid with a molecular weight of 385.535 Da. To investigate the effect of DCZ0858 on DLBCL cell lines, seven cell lines, OCI-LY8, NU-DUL-1, OCI-LY1, SUDHL-4, DB, TMD8, and U2932, were selected for this study. First, when treating these cells with different concentrations of DCZ0858 (2.5, 5, 10, 20, and 40 μM) for 48 h, the resulting IC_50_ (half-maximal inhibitory concentration) values were as follows: 14.4 μM (DB), 9.7 μM (TMD8), 8.8 μM (U2932), 11.5 μM (SUDHL-4), 7.4 μM (OCI-LY8), 10.1 μM (OCI-LY1), and 10.7 μM (NU-DUL-1) (Fig. 1b). Thus, DCZ0858 exerted an obvious inhibitory effect on these DLBCL cell lines. On the one hand, as shown in Fig. 1c, the IC_50_ decreased with increasing DCZ0858 concentration and treatment time, indicating concentration-dependent and time-dependent effects of DCZ0858 in the DLBCL cell lines. Notably, low toxicity was observed in the normal human peripheral blood mononuclear cells (PBMCs) and human B lymphocytes (WIL2-S), which were both treated with DCZ0858 (10, 20, and 40 μM) (Fig. 1d). On the other hand, the colony formation experiment exhibited the same trend, with an inverse correlation observed between the drug concentration and the number of colonies (Fig. 1e).

### DCZ0858 induces cell cycle arrest in the DLBCL cells studied

**Fig. 2:**
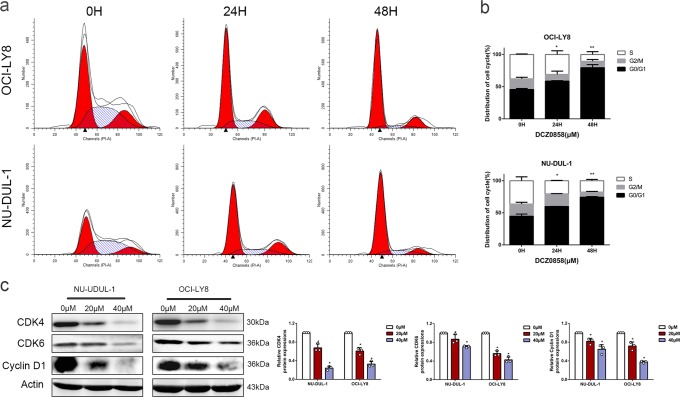
DCZ0858 promotes DLBCL cell cycle arrest in the G0/G1 phase. **a** NU-DUL-1 and OCI-LY8 cells were treated with 10 μM DCZ0858 for different times (24 and 48 h), stained with PI, and analyzed by flow cytometry. **b** Percentages of G0/G1, S, and G2/M phases in the cell cycle are shown in the statistical graph. **c** After 48 h of treatment with DCZ0858 (20 and 40 μM), the protein extracts from the NU-DUL-1 and OCI-LY8 cells were analyzed by western blotting to determine the changes in the levels of CDK6, CDK4, and cyclin D1 proteins. All data are shown as the means ± S.D. on the basis of triplicate measures; *P < 0.05 and **P < 0.01

Cell cycle arrest is a crucial mechanism by which anticancer drugs inhibit tumor growth. To address the influence of DCZ0858 on the cell cycle, we treated the OCI-LY8 and NU-DUL-1 cells with different concentrations of DCZ0858 (0 or 10 μM) for various times (0, 24, and 48 h) and analyzed them by flow cytometry. We found that the proportion of cells in the G0/G1 phase was significantly increased with increased duration in the presence of 10 μM DCZ0858 (Fig. 2a, b). We then examined the expression of cyclin D1, CDK4, and CDK6 proteins, which are all inhibitors of G0/G1 phase arrest. Treatment with DCZ0858 caused a significant decrease in these cell cycle-promoting proteins (Fig. 2c), a finding consistent with the DCZ0858-induced cell cycle arrest in the G0/G1 phase in the DLBCL cells.

### DCZ0858 induces cell apoptosis via caspase activation

**Fig. 3:**
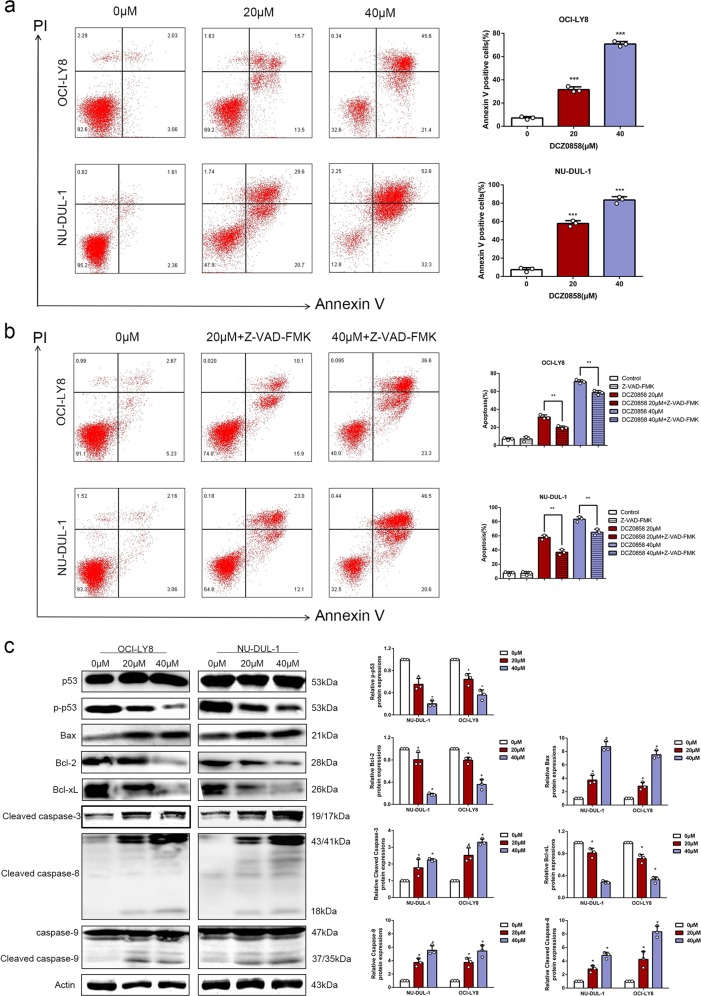
DCZ0858 induces cell apoptosis via caspase activation. **a** NU-DUL-1 and OCI-LY8 cells were treated with DCZ0858 for 48 h, double stained with annexin V-FITC/PI, and analyzed by flow cytometry. **b** NU-DUL-1 and OCI-LY8 cells were treated with Z-VAD-FMK and DCZ0858 for 48 h, and the cells were analyzed by flow cytometry. **c** Western blotting was used to analyze the expression levels of the caspase family proteins, namely, caspase-3, caspase-8, caspase-9, and the B-cell lymphoma family proteins (Bcl-2, Bax, and Bcl-xL). All values represent the mean ± SD of three sets of experimental data; *P < 0.05, **P < 0.01, and ***P < 0.001

Apoptosis is a physiological death program that can be experimentally induced by several regulatory factors. It has been reported that the malignancy of tumor cells is related to the inhibition of apoptosis. To determine whether apoptosis is associated with the DCZ0858-induced inhibition of tumor cell growth, OCI-LY8 and NU-DUL-1 cells were treated with 0, 20, and 40 μM DCZ0858 for 48 h. The results showed that DCZ0858 significantly increased tumor cell apoptosis in a dose-dependent manner (Fig. 3a). To further characterize the inhibitory effect of DCZ0858, Z-VAD-FMK, a pan-caspase inhibitor, was used to treat tumor cells in combination with DCZ0858 for 48 h. In the presence of the caspase inhibitor, the percentage of apoptotic cells was significantly reduced (Fig. 3b). Furthermore, apoptosis-related proteins were analyzed by western blotting. The levels of cleaved caspase-3, -8, -9, and Bax increased with drug concentration, while Bcl-2 and Bcl-xL levels decreased (Fig. 3c), demonstrating that DCZ0858-activated tumor cell apoptosis in a dose-dependent manner.

### The JAK2/STAT3 pathway is blocked by DCZ0858 in the DLBCL cells

**Fig. 4:**
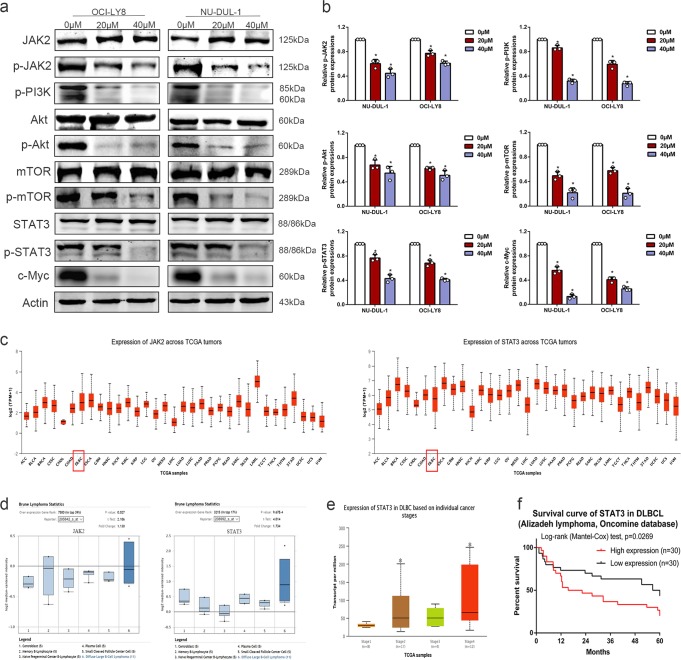
Mechanism of DCZ0858-induced tumor inhibition. **a** NU-DUL-1 and OCI-LY8 cells were treated for 48 h with DCZ0858 (20 and 40 μM), and then, the levels of JAK2, phospho-JAK2, PI3K, phospho-PI3K, Akt, phospho-Akt, mTOR, phospho-mTOR, STAT3, phospho-STAT3, c-Myc, and actin were assessed by western blotting. **b** The gray values for the protein bands represent the mean ± SD of three sets of experimental data. **c** The expression of JAK2 and STAT3 in a variety of malignancies from the TCGA database. **d** The expression of JAK2 and STAT3 in DLBCL patients in the Oncomine database. **e** The expression of STAT3 in DLBCL cells based on individual tumor stages. Additionally, upregulated STAT3 was associated with advanced cancer stages. **f** The survival curve of the DLBCL patients based on STAT3 expression in the Oncomine database; *P < 0.05

In malignancies, intracellular biochemical pathways are activated by a series of different proteins, and the dynamics of these proteins contribute to tumor progression. The development of effective therapeutic treatments may depend on the in-depth characterization of the protein interactions within these pathways. After treatment with different concentrations of DCZ0858 for 48 h, OCI-LY8 and NU-DUL-1 cell proteins were extracted and analyzed by western blotting. We tested a series of key regulatory factors involved in signal transduction in cell physiology and pathology, such as the JAK2/STAT3 pathway, p38/MAPK pathway, Erk pathway, JNK pathway, and NF-κB pathway (Supplementary Fig. S1 and Fig. 4a). The results showed that the protein levels of p-JAK2, p-PI3K, p-Akt, p-mTOR, p-STAT3, and c-Myc in the DCZ0858-treated cells decreased significantly in a dose-dependent manner, while the levels of total JAK2, PI3K, Akt, mTOR, and STAT3 were not affected (Fig. 4a, b). These results indicate that DCZ0858 may induce cell cycle arrest and activate cell apoptosis by inhibiting the JAK2/STAT3-signaling pathway.

To clarify the relationship between JAK2/STAT3 and DLBCL, we searched two public databases (TCGA and Oncomine) that are widely used in oncology research. According to the TCGA database, JAK2 and STAT3 had high expression levels in a variety of malignancies, including lymphoid neoplasm DLBC (Fig. 4c). The Oncomine database showed that both JAK2 and STAT3 were highly expressed in samples taken from DLBCL patients (Fig. 4d). Additionally, upregulated STAT3 was associated with advanced tumor stage, and patients with stage 4 tumors had higher STAT3 levels (Fig. 4e). Moreover, STAT3 was closely associated with the prognosis of DLBCL patients, and those with high STAT3 expression had poor overall survival (Fig. 4f). These results suggest that JAK2/STAT3 signaling is of value in the progression of DLBCL and that a compound (DCZ0858) that inhibits this signal may have a potential role in the treatment of DLBCL.

### The JAK2/STAT3 pathway is involved in DCZ0858-mediated antitumor effects in the DLBCL cells

**Fig. 5:**
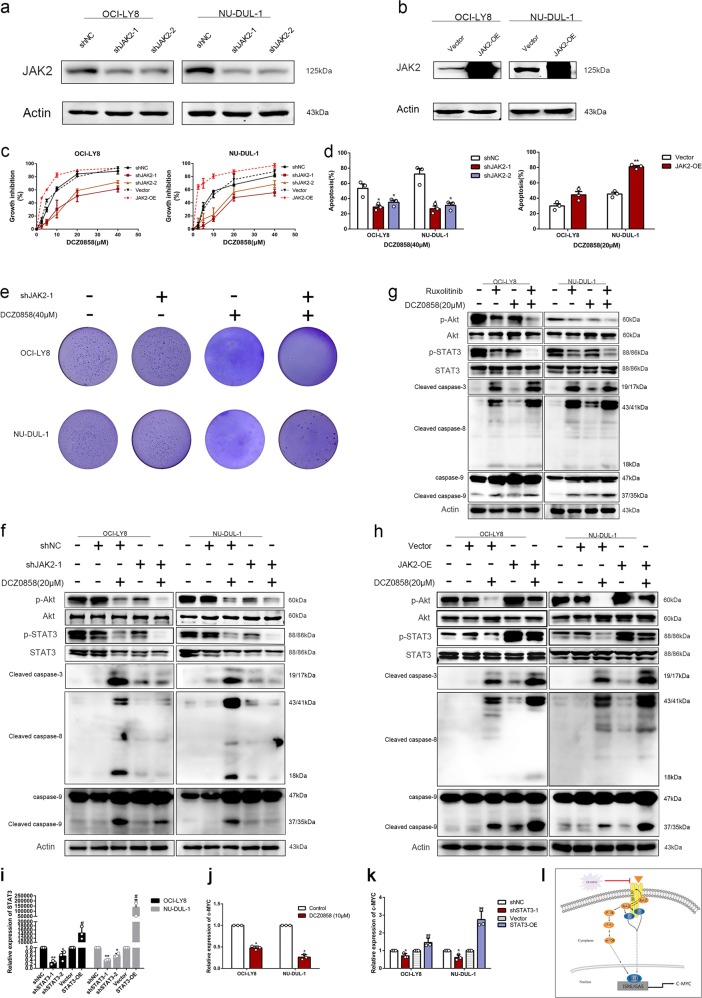
The JAK2/STAT3 pathway is involved in DCZ0858-mediated anti-tumor effects in the DLBCL cells. **a** The efficiency of JAK2 knockdown was verified by western blot analysis. **b** The efficiency of JAK2 overexpression was determined by western blotting. **c** OCI-LY8 and NU-DUL-1 cells were cotreated with JAK2 shRNAs and DCZ0858 (2.5–40 μM) for 48 h. CCK-8 assays were used to detect the inhibition rates at different DCZ0858 concentrations. **d** NU-DUL-1 and OCI-LY8 cells were cotreated with JAK2 shRNAs and DCZ0858 (40 μM) for 48 h, double-stained with annexin V-APC/7-AAD, and analyzed by flow cytometry. **e** Clone colonies formed by the OCI-LY8 and NU-DUL-1 cells cotreated with shJAK2-1 and DCZ0858 (40 μM); colonies in each well were quantified. **f** NU-DUL-1 and OCI-LY8 cells were cotreated with shJAK2-1 and DCZ0858 (40 μM) for 48 h and then protein levels were assessed by western blotting. **g** NU-DUL-1 and OCI-LY8 cells were pretreated with JAK2 inhibitor (ruxolitinib) for 2 h and then treated with DCZ0858 (40 μM) for 48 h. **h** NU-DUL-1 and OCI-LY8 cells were cotreated with JAK2-OE and DCZ0858 (40 μM) for 48 h, and analyzed by western blotting. **i** The efficiency of STAT3 knockdown and overexpression was verified by qRT-PCR. **j** The expression of c-Myc after treatment with DCZ0858. **k** The change in c-Myc expression after STAT3 overexpression and knockdown. **l** A schematic showing the involvement of JAK2/STAT3 in DCZ0858-mediated antitumor effects in the DLBCL cells. All data are presented as the means ± SD on the basis of triplicate measures; *P < 0.05, **P < 0.01, and #*P* < 0.05

To discern whether the JAK2/STAT3 pathway was involved in the cellular process affected by DCZ0858 treatment, we artificially downregulated and upregulated JAK2 expression using shRNA (shJAK2) and an overexpression vector (JAK2-OE) in the OCI-LY8 and NU-DUL-1 cells. The efficiency of JAK2 knockdown and overexpression was verified (Fig. 5a, b). Functional tests were also performed. Compared to the cells treated with DCZ0858 alone, JAK2-knockdown OCI-LY8 cells had significantly increased IC_50_ values after DCZ0858 treatment (Fig. 5c; IC_50_ of 22.3 μM for shRNA1 and 19.7 μM for shRNA2). The same trend was also observed for the DCZ0858-treated NU-DUL-1 cells (IC_50_ of 25.2 μM for shRNA1 and 21.5 μM for shRNA2). At the same time, JAK2 overexpression made cells more sensitive to DCZ0858, showing a lower IC_50_ concentration (Fig. 5c). Flow cytometry results showed that JAK2 knockdown decreased the apoptosis rate induced by DCZ0858, while more apoptotic cells were found in a group of cells with combined DCZ0858 and JAK2 overexpression, suggesting that JAK2 was involved in DCZ0858-induced apoptosis of DLBCL cells (Fig. 5d). In addition, JAK2 knockdown partly weakened the inhibitory effect induced by DCZ0858 on colony formation units in the DLBCL cells (Fig. 5e).

Interestingly, after JAK2 knockdown, downstream signaling was blocked in a manner similar to that upon treatment with DCZ0858 alone, with a decrease in the phosphorylation levels of these downstream proteins (Fig. 5f). However, when cells were treated with DCZ0858 and JAK2 shRNA in combination, although the signaling pathway was blocked, the expression of pro-apoptotic proteins was lower than that of the group treated with DCZ0858 alone (Fig. 5f). However, strengthened antitumor effects were obtained when the cell were cotreated with DCZ0858 and the JAK2 inhibitor ruxolitinib (Fig. 5g). To further study the relationship between JAK2 and DCZ0858, a group of cells cotreated with the JAK2 overexpression vector and DCZ0858 was established. The results showed that, in the JAK2-overexpressing cells, DCZ0858 promoted the expression of caspase-3, -8, and -9 to a significantly greater level, implicating JAK2 as a potential functional target of DCZ0858 (Fig. 5h). In addition, we also constructed STAT3 knockdown and overexpression plasmids (Fig. 5i). Similar to the effect of DCZ0858 on c-Myc, silenced STAT3 also downregulated c-Myc expression at the transcription level (Fig. 5j, k) and thus partially exhibited the DCZ0858-mediated tumor suppression phenotype.

### DCZ0858 treatment inhibits tumor growth in a nude mouse model

**Fig. 6:**
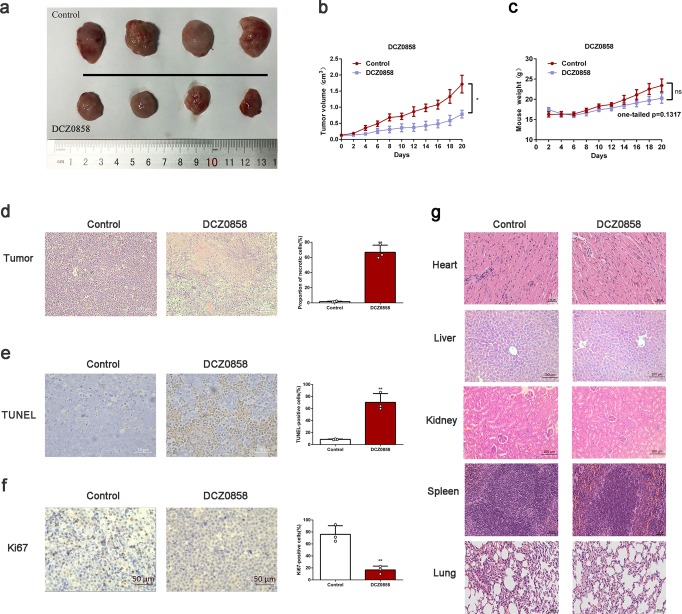
DCZ0858 inhibited tumor growth in vivo. **a** Tumor specimens photographed with a high-definition digital camera. **b** Tumor volume was measured every 2 days. **c** The weight of the mice was recorded every 2 days. **d** H&E staining of the control and DCZ0858-treated transplanted tumor tissues in the nude mice (original magnification: ×400); **e** Apoptotic cells from the xenograft tumors in the nude mice as detected by TUNEL staining (magnification: ×400). **f** Ki67 staining of the control and DCZ0858-treated xenograft tumor tissues (original magnification: ×400). **g** H&E staining of the control and DCZ0858-treated heart, liver, kidney, spleen, and lung tissues in the nude mice (original magnification: ×200); *P < 0.05 and **P < 0.01

To further investigate the inhibitory effect of DCZ0858 on tumor growth and determine any possible toxic side effects, we established an animal xenograft model using human DLBCL cells (OCI-LY8). We discovered that DCZ0858 significantly inhibited in vivo tumor growth (Fig. 6a, b). The results of H&E staining showed that, in mice treated with DCZ0858, the area of tumor necrosis was significantly greater than it was in untreated mice (Fig. 6d). In addition, in the DCZ0858-treated group, the proportion of apoptotic cells was also significantly increased, as assessed by TUNEL staining (Fig. 6e), while that of proliferating cells was markedly decreased, as shown by staining for the proliferation marker Ki67 (Fig. 6f). However, mouse body weight remained substantially unchanged throughout these experiments (Fig. 6c). Moreover, after DCZ0858 treatment, no significant differences between the two groups were observed in the heart, liver, kidney, spleen, and lung tissues of mice, suggesting that DCZ0858 had no obvious toxic effect on some important organs (Fig. 6g). Thus, we can conclude that DCZ0858 is a nontoxic and promising drug for the treatment of DLBCL.

## Discussion

DLBCL is one of the most common lymphoid neoplasms, representing 30–58% of all NHL. In addition to lymph nodes, <40% of all tumors originate in extranodal sites.^13^ Although intensified chemotherapy with R-ACVBP (rituximab, doxorubicin, cyclophosphamide, vindesine, bleomycin, and prednisone) plus standard R-CHOP significantly improves the survival of patients with DLBCL, according to the International Prognostic Index, the high heterogeneity in molecular pathogenesis and clinical presentation still poses a major therapeutic challenge.^14,15^

Due to the low cytotoxicity induced in normal cells, wide therapeutic range, and highly efficient tumor inhibition, small molecular compounds are being extensively investigated as anticancer drugs. Our previous study showed that the novel small molecular inhibitor DCZ0858 induces antitumor effects in multiple myeloma and overcomes the protection provided by the bone marrow microenvironment via the dual inhibition of mTOR1/2.^16^ In the present study, we found that a novel silicone derivative of osalmid, DCZ0858, exerted a concentration-dependent antiproliferative effect on seven DLBCL cell lines. Interestingly, although both the time- and concentration-dependent inhibitory effects were observed in NU-DUL-1 and OCI-LY8 cells, as shown by the cell proliferation assay results, these two cell lines showed only concentration-dependent effects in the flow cytometry analysis. This discrepancy is likely due to the activation of DCZ0858-induced nonapoptotic cell death pathways, such as autophagy and ferroptosis. Detailed exploration in future studies is necessary.

For most malignancies, impaired apoptosis is the leading cause of radioresistance and chemoresistance, and moderate modulation of apoptosis-associated signaling pathways is one of the most promising strategies for cancer therapy.^17^ The death receptor pathway (extrinsic) and the mitochondria-mediated pathway (intrinsic) are the two major apoptosis signaling pathways. In the extrinsic pathway, activated initiator caspase-8 cleaves the downstream effector caspase-3, ultimately leading to the morphological changes typical of apoptosis.^18^ In contrast, the intrinsic pathway is triggered by p53-induced Bcl-2 attenuation and Bax activation along with the subsequent cleavage of caspase-9.^19^ In the current study, the flow cytometry data showed that DCZ0858 promoted cell apoptosis, a finding that was confirmed by a rescue experiment with the caspase inhibitor Z-VAD-FMK. In addition, DCZ0858 activated both internal and external apoptotic pathways, as manifested through the activation of caspase-8, -3 and caspase-9 and the silencing of the p53, Bcl-2, and Bcl-xL proteins.

Cell cycle regulation consists of several checkpoints, most of which are associated with the sequential activation of cyclin-dependent kinases (CDKs). The activation of cyclin D1, which subsequently interacts with CDK4 or CDK6, leads to the inactivation of the restrictive G1 checkpoint, suppressing cell cycle arrest.^20^ In our study, DCZ0858 induced cell cycle arrest in the G0/G1 phase and inhibited cell transition into the S phase. In addition, DCZ0858 treatment decreased the expression of some key cell cycle regulatory proteins (cyclin D1, CDK4, and CDK6), which confirmed the role of DCZ0858 in inducing cell cycle arrest in the G0/G1 phase.

Both the tyrosine kinase JAK and the cytosolic transcription factor STAT are composed of several subunits, and activation of JAK and STAT proteins through the phosphorylation of specific tyrosine residues is closely associated with tumor progression.^21,22^ In the current study, the decreased levels of p-JAK2 and p-STAT3 following treatment with DCZ0858 provided insights into the antitumor effects of this novel agent in DLBCL cells. Interestingly, mTOR was reported to directly upregulate STAT3 activity by enhancing its phosphorylation at the S727 residue.^23^ Alternative approaches, including JAK and mTOR inhibition, could suppress STAT3 activity and function as therapeutic methods for lymphoproliferative disorders caused by constitutive STAT3 activation.^24^ In our study, we explored the activation state of possible factors involved in the JAK2/STAT3 pathway and found that DCZ0858 inhibited the phosphorylation of PI3K, Akt, and mTOR, which most likely contributed to the observed antitumor effects. To identify the possible target of DCZ0858, we artificially downregulated JAK2 expression in the DLBCL cells. Although the signaling pathway remained blocked, JAK2 knockdown partly abrogated the DCZ0858-mediated tumor-suppressive effect on cell proliferation and apoptosis. However, the combination of the JAK2 inhibitor and DCZ0858 exerted a stronger inhibitory effect on both the signaling pathways and the cell malignant phenotypes (proliferation and apoptosis). These differences could be explained as follows: DCZ0858 is regarded as another inhibitor of JAK2, and therefore, combining the two inhibitors showed a synergistic effect, and JAK2 knockdown led to the reduction of JAK2 expression at the transcription level; thus, abrogating the target site made DCZ0858 less effective.

Finally, the animal model data provided another rationale for the potential use of DCZ0858 in DLBCL treatment. Intravenous administration of DCZ0858 significantly prevented tumor growth without affecting body weight or causing any damage to important organs, indicating that DCZ0858 was well tolerated in the mice and did not elicit major toxic effects. Tissue staining and immunohistochemical analysis confirmed that DCZ0858 weakened cell proliferation and enhanced cell apoptosis, thereby inhibiting tumor growth in the xenograft models.

In summary, the antitumor effect of DCZ0858 on the DLBCL cells was demonstrated in this study both in vitro and in vivo. DCZ0858 induced cell apoptosis and cell cycle arrest in the G0/G1 phase by inactivating the JAK2/STAT3 pathway. Our study implied that DCZ0858 might be a novel therapeutic tool for the treatment of DLBCL patients. However, further research is necessary to clarify the precise cellular mechanism of DCZ0858 action and to demonstrate its true clinical value.

## Materials and methods

### Cells and cell culture

The OCI-LY1, OCI-LY8, and NU-DUL-1 human cell lines were kindly provided by Prof. Xiaoyan Zhou, a chief physician in the Department of Pathology, Fudan University Shanghai Cancer Center (Shanghai, China). U2932 and TMD8 cells were gifts from Prof. Dongsheng Xu (Shanghai Tenth People’s Hospital, Tongji University of Medicine, Shanghai, China). Two other cell lines, SUDHL-4 and DB, were purchased from the American Type Culture Collection (Manassas, VA, USA). Human PBMCs were obtained from two men and one woman (aged 25, 27, and 28 years, respectively). These donors were volunteers and provided informed consent in advance of sample collection. The OCI-LY1 and OCI-LY8 cells, belonging to the GCB subtype of DLBCL, were cultured with Iscove’s modified Dulbecco’s medium (Gibco, Waltham, MA, USA) supplemented with 10% fetal bovine serum (FBS; Gibco, Thermo Fisher Scientific, Inc.) and 1% penicillin–streptomycin (Gibco, Thermo Fisher Scientific, Inc.). The other four cell lines were cultured in RPMI 1640 medium (Gibco, Waltham, MA, USA) containing 10% FBS and 1% penicillin–streptomycin. All cells were cultured in a 37 °C incubator with 5% carbon dioxide.

### Reagents and antibodies

A DCZ0858 stock solution (40 mM) was prepared in dimethyl sulfoxide (DMSO; Sigma, St. Louis, MO, USA) according to the relative molecular mass and stored at −20 °C in the dark. A Cell Counting Kit-8 (CCK-8) was obtained from Shanghai Yeasen Biotechnology Co., Ltd. (Shanghai, China). The Annexin V/PI used for apoptosis staining and propidium iodide (PI) used for staining permeable cells were purchased from BD Pharmingen (San Diego, CA, USA). The pan-caspase inhibitor Z-VAD-FMK was supplied by Selleck Chemicals (Houston, TX, USA). Antibodies against JAK2, phospho-JAK2, phospho-PI3K, Akt, phospho-Akt, STAT3, phospho-STAT3, cleaved caspase-8, Bax, B-cell lymphoma-2 (Bcl-2), Bcl-xL, actin, and c-Myc were purchased from Cell Signaling Technology (Beverly, CA, USA). Cleaved caspase-3, caspase-9, phospho-p53, p53, CDK4, CDK6, cyclin D1, mTOR, and p-mTOR were purchased from Abcam (Cambridge, UK).

### Lentivirus packaging and cell transfection

sh-JAK2 and sh-STAT3 plasmids were synthesized by Bioegene Co., Ltd. (Shanghai, China) as previously described.^25^ The RNAi lentiviral vector CMV-copGFP-T2A-puro-H1-shRNA was constructed by Shanghai Bioegene Co., Ltd. The RNAi lentiviral vector and plasmid were cotransfected into 293T cells using Lipofectamine 3000 (Thermo Fisher Scientific, Waltham, MA, USA). After culturing for 48 h, the supernatant was concentrated and collected. The packaged recombinant lentivirus was then used for JAK2 knockdown in OCI-LY8 and NU-DUL-1 cells. After 3 days of cell transfection, GFP expression in the cells was observed using an inverted fluorescence microscope. The overexpression vectors JAK2 and STAT3 were designed by Vigene Biosciences Corporation (Rockville, MD, USA).

### RNA extraction and qRT-PCR assays

Total RNA was extracted using an RNA-Quick purification kit (ES Science, Shanghai, China). Then, the RNA was reverse transcribed to cDNA using the PrimeScript RT reagent kit with gDNA Eraser (TaKaRa Bio Inc., Shiga, Japan) according to the manufacturer’s instructions. The amplification procedures were performed on an ABI 7000 PCR detection system (ABI, Vernon, CA, USA). We used the relative quantitative (2^−ΔΔCt^) method to calculate the relative expression level of JAK2. In view of its higher stability and consistency, glyceraldehyde 3-phosphate dehydrogenase (GAPDH) was used as the internal control to normalize the RNA input. The sequences of the primers used in this study are shown in Table 1.

**Table 1:** The sequences of primers used in qRT-PCR

Gene	Sequences
JAK2	Forward: 5′-TCTGGGGAGTATGTTGCAGAA-3′
	Reverse: 5′-AGACATGGTTGGGTGGATACC-3′
STAT3	Forward: 5′-ACCAGCAGTATAGCCGCTTC-3′
	Reverse: 5′-GCCACAATCCGGGCAATCT-3′
c-Myc	Forward: 5′-GTCAAGAGGCGAACACACAAC-3′
	Reverse: 5′-TTGGACGGACAGGATGTATGC-3′
GAPDH	Forward: 5′-GGAGCGAGATCCCTCCAAAAT-3′
	Reverse: 5′-GGCTGTTGTCATACTTCTCATGG-3′

### Cytotoxicity assay

DLBCL cells (2 × 10^5^ cells/ml) were seeded into 96-well plates at a volume of 95 μl/well and then treated with 5 μl/well of DCZ0858 at different concentrations (2.5, 5, 10, 20, and 40 μM) for 48 h. OCI-LY8 and NU-DUL-1 cells were treated for an additional 24 and 72 h to explore the time dependence of the drug effects. PBMCs were plated at a density of 4 × 10^5^/ml. At the time of the reaction, 10 μl of CCK-8 kit reagent was added to each well and incubated at 37 °C for 2 h. The final absorbance was measured at a wavelength of 450 nm with a microplate reader.

### Cell cycle analysis

The OCI-LY8 and NU-DUL-1 cells were pretreated in serum-free medium for 12 h and then mixed with complete medium to form a cell suspension with a density of 2 × 10^5^/ml. The cell suspension was seeded into a 12-well plate and treated with 10 μM DCZ0858 for 0, 24, and 48 h. The cells were collected, washed with phosphate-buffered saline (PBS), fixed in precooled 75% ethanol, and stored at −20 °C overnight. Finally, after washing with PBS, the cell/alcohol mixture was stained with propidium iodide (PI) (BD) for 15 min at room temperature and analyzed by flow cytometry.

### Cell apoptosis detection

The OCI-LY8 and NU-DUL-1 cells were seeded at a density of 2 × 10^5^/ml into 12-well plates and treated with DCZ0858 (0, 20, and 40 μM) and/or Z-VAD-FMK (50 μM). After 48 h incubation in a 37 °C incubator containing 5% carbon dioxide, Annexin V-fluorescein isothiocyanate (FITC)/propidium iodide (PI) was used for cell staining at 4 °C for 30 min in the dark, followed by PI staining for 10 min. Apoptotic cells were mainly composed of early (Annexin V+/PI−) and late (Annexin V+/PI+) apoptotic cells, based on the flow cytometry results. In cells that were transfected with lentivirus, Annexin V-APC/7-AAD was used to stain the cells.

### Plate colony formation assay

Complete medium and 3.5% agar were mixed at a 5:1 ratio to form the substratum of a six-well plate, while 1.66% agar was mixed at a 1:5 ratio with NU-DUL-1 and OCI-LY8 cells (pretreated with DCZ0858 at 0, 20, or 40 μM) as a superstratum. After 2 weeks, 0.5% crystal violet was added to completely infiltrate the gel and incubated for 30 min at room temperature.

### Western blot analysis

After the NU-DUL-1 and OCI-LY8 cells were treated for 48 h with DCZ0858 (20 and 40 μM), the cytosolic proteins were extracted using lysis buffer (100 mM Tris–HCl, pH 6.8; 4% SDS; and 20% glycerol). Sodium dodecyl sulfate–polyacrylamide gel electrophoresis (SDS–PAGE) with 8–15% gels was used to isolate cytosolic proteins (30 μg per lane). The cells were transferred to nitrocellulose or polyvinylidene fluoride membranes, which were then blocked with 5% nonfat milk or 5% bovine serum albumin (BSA) for 1 h at room temperature. After incubation for 4 h at 4 °C with the primary antibody, the membranes were washed three times with Tween 20 (1:1000 dilution)–PBS for 10 min each. Finally, the membranes were treated with the appropriate secondary antibody (anti-rabbit or anti-mouse IgG) at room temperature for 1 h and then the bands were measured by an Odyssey two-color infrared laser imaging system (LICOR, Lincoln, NE, USA).

### Xenograft model construction

Ten 6-week-old nude male mice purchased from the Shanghai Laboratory Animal Center (SLAC, Shanghai, China) were selected as tumor xenograft models. The mice were housed in a standard laboratory and fed standard food during the experiment. A suspension of 3 × 10^7^ OCI-LY8 human cells per milliliter was prepared in serum-free medium, and 100 μl of the suspension was subcutaneously injected into the upper arm of each mouse. When the tumor volume was an appropriate level (100 mm^3^), the mice were randomly assigned to the control or DCZ0858 group (*n* = 2/group). The DCZ0858 group was intraperitoneally injected with DCZ0858 (10 mg/kg every 2 days) for 20 days, while the control group was simultaneously injected with the same volume of normal saline. Tumor sizes and mouse body weight were measured every 2 days until the end of the experiment. Tumor volume was obtained by the following formula: *V* = *A* × *B*^2^/2 (*A* = longest diameter; *B* = smallest diameter). Tumors, livers, and kidneys were stained with H&E, TUNEL, and a Ki67-specific stain. This animal experiment was approved by the Shanghai Science and Technology Commission (ID: SYXK 2011-0111).

### Statistical analysis

The data are presented as the means ± standard deviation (SD). Student’s *t*-test was used for determining significance of intergroup data, while the significance of multiple comparisons was assessed by one-way ANOVA. The statistical significance was assessed using SPSS v20.0 statistical analysis software (IBM Corp., Armonk, NY, USA), and ImageJ software (NIH, Bethesda, MD, USA) was used to quantify the western blot bands and cells positive for immunohistochemical staining. The IC_50_ values were calculated by CalcuSyn software 2.0 (Biosoft, Cambridge, UK). The data in the TCGA database were analyzed based on the UALCAN website.^26^ A *P* < 0.05 was considered statistically significant.

## Supplementary information

Supplementary Materials
