# Supplementary material for: A novel silicone derivative of natural osalmid (DCZ0858) induces apoptosis and cell cycle arrest in diffuse large B-cell lymphoma via the JAK2/STAT3 pathway
Source: Signal Transduct Target Ther. 2020 Apr 1;5:31. doi: 10.1038/s41392-020-0123-0 (PMC7118088; doi:10.1038/s41392-020-0123-0)
Supplement: Supplementary file 1 — Supplementary Materials [file 41392_2020_123_MOESM1_ESM.docx]

Supplementary Materials for

A Novel Silicone Derivative of Natural Osalmid (DCZ0858) Induces Apoptosis and Cell Cycle Arrest in Diffuse Large B Cell Lymphoma via the JAK2/STAT3 Pathway

Kang Lu1,3, #, Bo Li2,#, Hui Zhang1,#, Zhijian Xu2, Dongliang Song1, Lu Gao1, Haiguo Sun2, Liping Li1, Yingcong Wang1, Qilin Feng1, Gege Chen1, Liangning Hu1, Rong Wei1, Yongsheng Xie1, Dandan Yu1, Xiaosong Wu1, Weiliang Zhu2,*, Jumei Shi1,*

^1^ Department of Hematology, Shanghai Tenth People’s Hospital, Tongji University, School of Medicine, Shanghai 200072, China;

^2^ CAS Key Laboratory of Receptor Research, Drug Discovery and Design Center, Shanghai Institute of Materia Medica, Chinese Academy of Sciences, Shanghai 201203, China;

^3^ Medical School of Nantong University, #19 Qixiu Road, Nantong 226001, China.

Correspondence to: Jumei Shi ([shijumei@tongji.edu.cn](mailto:shijumei@tongji.edu.cn)) or Weiliang Zhu (wlzhu@simm.ac.cn).

**This PDF file includes:**

Figures. S1 to S2


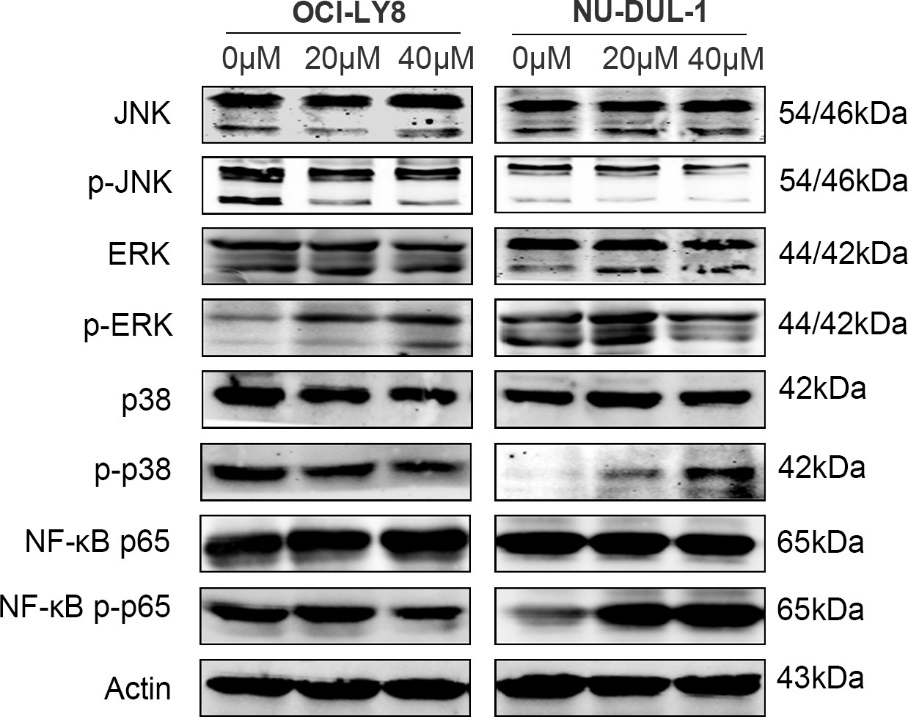


Figure. S1. Exploration of other signal pathways regulated by DCZ0858.

After treatment with DCZ0858 (20 and 40μM) for 48 h, cell pellets were collected for western blot detection.


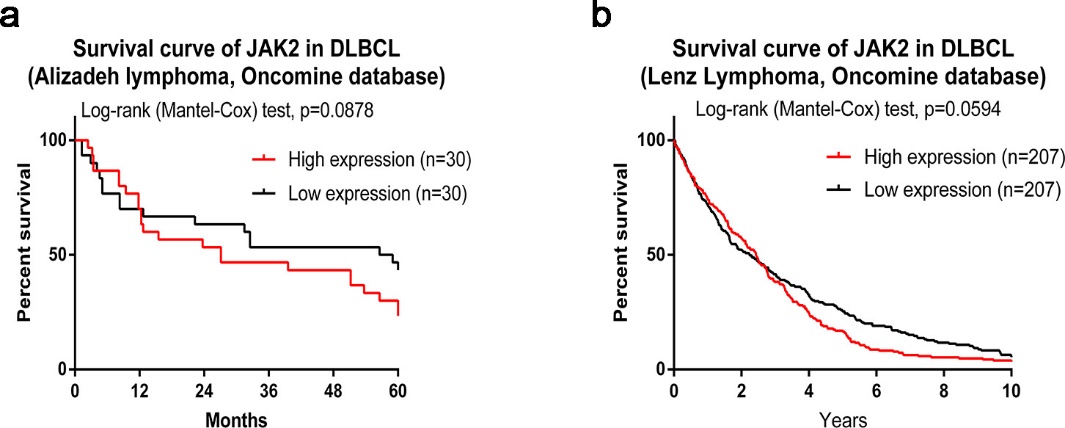


Figure. S2. The survival curve of DLBCL patients based on JAK2 expression in Oncomine database.

(a) Overall survival status of DLBCL patients in Alizadeh lymphoma (n=60). (b) Overall survival status of DLBCL patients in Lenz lymphoma (n=414).
